# The skeletal ontogeny of *Astatotilapia burtoni* – a direct-developing model system for the evolution and development of the teleost body plan

**DOI:** 10.1186/s12861-018-0166-4

**Published:** 2018-04-03

**Authors:** Joost M. Woltering, Michaela Holzem, Ralf F. Schneider, Vasilios Nanos, Axel Meyer

**Affiliations:** 1 Chair in Zoology and Evolutionary Biology, Department of Biology, University of Konstanz, Universitätsstraße 10, 78457 Constance, Germany; 2 Current address: Department of Biological an Medical Sciences, Oxford Brookes University, Headington Campus, Oxford, OX3 0 BP UK

**Keywords:** Direct-development, Indirect-development, *Astatotilapia burtoni*, Embryonic development, Axial skeleton, Appendicular skeleton, Evo-devo, Fin, Teleost

## Abstract

**Background:**

The experimental approach to the evolution and development of the vertebrate skeleton has to a large extent relied on “direct-developing” amniote model organisms, such as the mouse and the chicken. These organisms can however only be partially informative where it concerns secondarily lost features or anatomical novelties not present in their lineages. The widely used anamniotes *Xenopus* and zebrafish are “indirect-developing” organisms that proceed through an extended time as free-living larvae, before adopting many aspects of their adult morphology, complicating experiments at these stages, and increasing the risk for lethal pleiotropic effects using genetic strategies.

**Results:**

Here, we provide a detailed description of the development of the osteology of the African mouthbrooding cichlid *Astatotilapia burtoni*, primarily focusing on the trunk (spinal column, ribs and epicentrals) and the appendicular skeleton (pectoral, pelvic, dorsal, anal, caudal fins and scales), and to a lesser extent on the cranium. We show that this species has an extremely “direct” mode of development, attains an adult body plan within 2 weeks after fertilization while living off its yolk supply only, and does not pass through a prolonged larval period.

**Conclusions:**

As husbandry of this species is easy, generation time is short, and the species is amenable to genetic targeting strategies through microinjection, we suggest that the use of this direct-developing cichlid will provide a valuable model system for the study of the vertebrate body plan, particularly where it concerns the evolution and development of fish or teleost specific traits. Based on our results we comment on the development of the homocercal caudal fin, on shared ontogenetic patterns between pectoral and pelvic girdles, and on the evolution of fin spines as novelty in acanthomorph fishes. We discuss the differences between “direct” and “indirect” developing actinopterygians using a comparison between zebrafish and *A. burtoni* development.

## Background

Teleost fishes are undoubtedly amongst the most diverse of vertebrates [1–4]. It therefore may seem paradoxical that the contribution of the fish model systems to the understanding of the development and evolution of the natural diversity of vertebrate body plans is relatively minor. This observation is made while acknowledging the enormous contribution of the model fish species zebrafish and medaka to the understanding of the processes of early development, such as germ layer induction, gastrulation and the subsequent formation of the anterior-posterior body axis (e.g. [5–9]). However, anatomical structures that develop later during development have remained surprisingly underexposed compared to for instance mammals. Where we have a very good understanding of the genetic networks underlying the differentiation and patterning of the axial and appendicular skeleton in mice, and the potential consequences of the evolution of these networks for changes in the body plan (e.g. [10–17]), there is less experimental evidence regarding the orthologous networks from fish. In many aspects this goes beyond a mere confirmation of the conservation of deeply homologous gene networks, but concerns essential questions regarding ancestral vertebrate characters that have disappeared from tetrapods, or novelties that arose in the fish lineages. Open questions in this field for instance relate to the transcription factor code that determines the positioning of median fins, the differentiation of dermal versus endochondral ossifying fin elements (including the secondary loss of the dermal part in tetrapods), and the further diversification of the fins and scale skeleton in teleosts.

One restraining factor appears related to the choice of fish model systems. Embryologists frequently make the distinction between “direct” and “indirect” developing organisms [18, 19]. The term “direct-development” is used for organisms whose embryos develop directly with adult features (i.e. resembling miniature adults). Examples of such species are amniotes, such as the mouse and the chicken. “Indirect-developing” organisms pass through a prolonged free-living and feeding larval stage that only partially resembles the adult [4, 18, 19]. The typical example of indirect-development is as occurs in frog species that know a tadpole stage as part of their life history [20, 21]. This type of development is characteristic for many anamniotes, and indirect-development occurs in many gradations, and also may differ for different characters within a given species [4, 18, 19]. In ray-finned fish (actinopterygians), the occurrence of indirect or direct modes of development is strongly related to the life history strategy of the species, and the presence of a distinct larval stage (also see the discussion section) [18, 19]. Direct-developing species usually have large yolk rich eggs, and the embryo completes its development while living off the maternal yolk supply, transforming directly into a feeding juvenile (i.e. a “definitive phenotype”) [18, 19]. Indirect-developing species usually have sparser yolk supply, and the onset of feeding precedes the completion of the body plan. In these species, development is only completed during a free feeding larval stage before transformation into a juvenile fish occurs [18, 19].

The most frequently used fish model system, zebrafish (*Danio rerio*), follows a typical pattern of indirect-development, with many adult features, such as the dermal skeleton and large parts of the trunk endoskeleton, only developing after several weeks of larval life [22–25]. The development of medaka (*Oryzias latipes*) is somewhat more direct, with a developed caudal fin present at hatching, but the other median fins appear only after several weeks in swimming and feeding larvae [23]. Such indirect-development brings a number of complications to the study of the development of adult characters. For genetic strategies, it is relevant that most developmental genes are involved in several biological processes (pleiotropy). Only very few zebrafish mutants identified in screens for embryonic characters [6] do survive until adulthood [7, 8]. For instance, one developmental bottleneck that has been reported in mutant zebrafish seems related to impaired swim bladder development [6, 26, 27]. This defect becomes lethal around the start of feeding stages, and prevents these fish from developing adult traits [28]. Forward genetic screens focusing on adult phenotypes will per definition circumvent this problem for the mutated genes identified [7, 8]. However, this strategy will not detect genes whose early loss of function is not compatible with the formation of a coherent organism that is able to perform tasks related to feeding, locomotion and cognition. As important transcription factors and signal transduction pathways are used repeatedly during development [29, 30], focusing on viable adult mutant phenotypes will bias towards genes primarily involved in restricted terminal differentiation events, omitting numerous genes that contribute fundamentally to the establishment of the *Bauplan.* A further complication related to indirect-development is that growth amongst free feeding larvae varies strongly [22, 25, 31], making it difficult to accurately predict development and to obtain synchronized series of material. Therefore, a direct-developing fish model system, i.e. a species that shows a rapid development from egg to juvenile, without relying on an extended free-swimming larval period, would be a welcome addition to the field. This is particularly relevant where it concerns the understanding of adult characters of the fish body plan that may otherwise remain partially inaccessible. Here, we describe the development of the skeleton of the *Haplochromine* mouthbrooding cichlid *Astatotilapia burtoni*, with particular focus on the post-cranial parts. Mouthbrooding species are known to generally have a more direct development than free spawning species [18], and we show that this species develops an adult body plan and skeleton within 2 weeks after fertilization, without the presence of a specialized larval period.

## Results

**Fig. 1:**
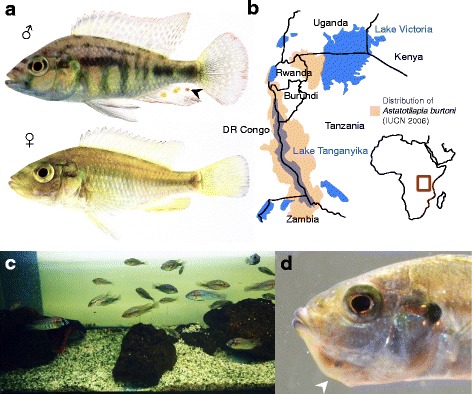
*Astatotilapia burtoni* morphology, distribution and husbandry. **a** *A. burtoni* shows pronounced sexual dimorphism, with colorful dominant males with their characteristic egg spots on the anal fin (black arrowhead), and plain females. **b** *A. burtoni* is native to Lake Tangayika in the East African Rift Valley, with additional, introduced populations western of Lake Victoria (distribution after IUCN 2006, geographical boundaries drawn after Google earth 2017). **c** Typical tank set up for *A. burtoni* to house a breeding group of 20–40 adult fish. Aquaria do not need to be planted but are best decorated with gravel and numerous rocks and hiding places, such as upturned half-flowerpots, which the fish will use during their mating rituals. **d** Mouthbrooding females can be recognized by their expanded lower jaw, leading to a pronounced “chin” in profile view (white arrowhead)

*A. burtoni* belongs to the radiation of mouthbrooding East-African cichlids [32–34], which are best known for their spectacular diversity and rapid evolution [35]. This species originates from lake Tanganyika, with additional, introduced populations present in the western tributaries to lake Victoria (Fig. 1a, b) [36]. *A. burtoni* is easy to maintain under laboratory conditions, and in our animal facility it spawns more reliably and can be kept more conveniently in relatively small tanks (100–180 l) than the better-known Nile tilapia (*Oreochromis niloticus*) (Fig. 1c).

*A. burtoni* exhibits strong sexual dimorphism, with colorful dominant males and plain females (Fig. 1a). Males are characterized by the “egg spots” on the anal fin, which play an important function in the mating ritual of *Haplochromine* cichlids, functioning as egg dummies [33, 37, 38]. During mating, the female deposits her eggs and takes them up in her mouth, after which the male fertilizes them while she approaches his egg spots [32, 39]. Mating usually takes place in the early morning hours, takes 5–15 min and its occurrence is frequently only noticed subsequently through the presence of mouthbrooding females, which can be recognized by the angular protrusion of their lower jaw, forming a pronounced “chin” (Fig. 1d). Embryos can be collected by gently opening the female’s mouth inside a container filled with aquarium water, and letting them drop out by gravity. Females of which eggs were removed typically spawn again within 2 weeks.

### General overview of *A. burtoni* embryonic development

**Fig. 2:**
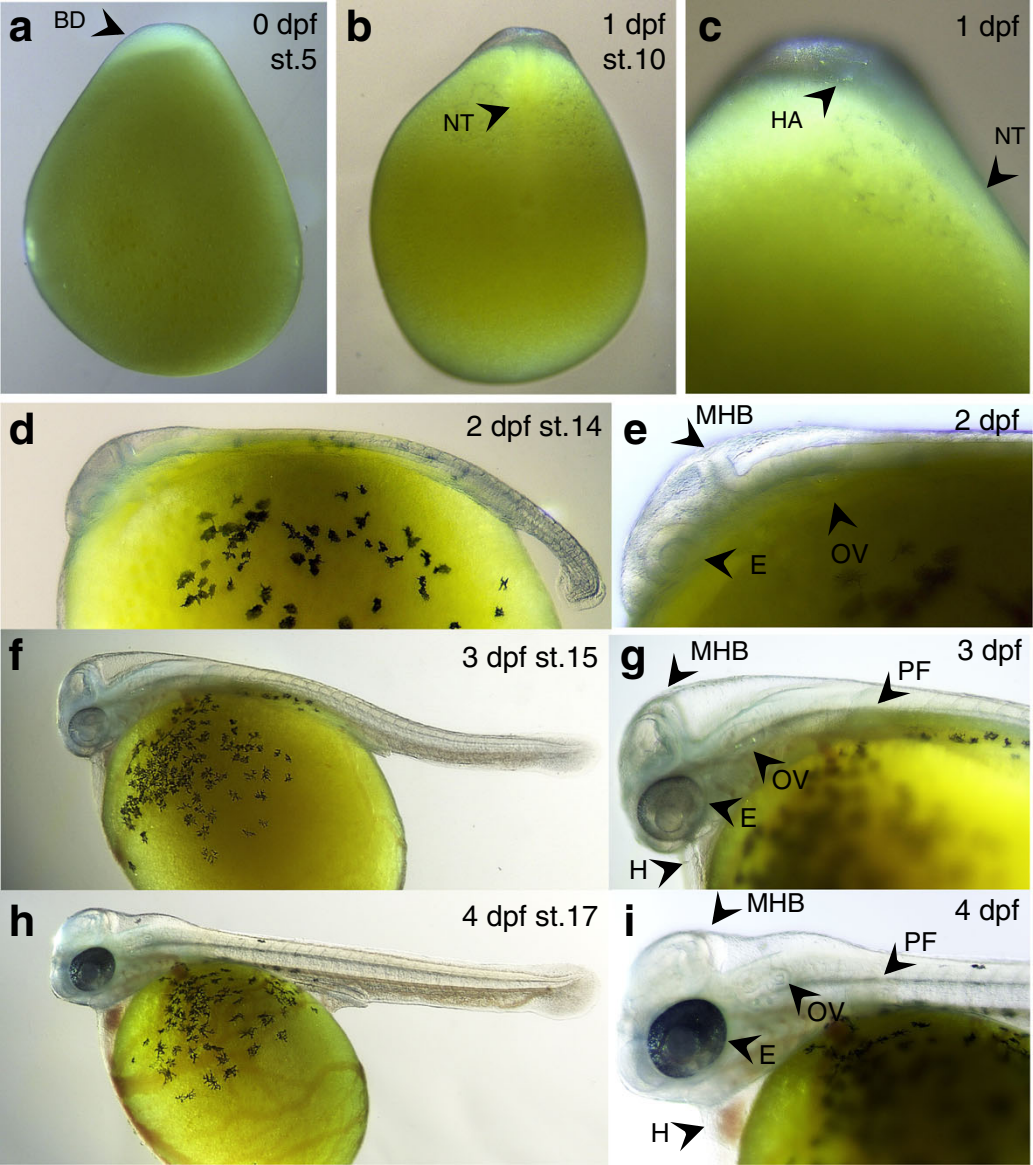
Early embryonic development of *A. burtoni*. **a** On the day of fertilization (0 dpf) embryos develop until gastrulation – (shown is a blastula stage embryo, ~ stage 5). As in other teleosts, embryos develop on top of the yolk. **b**, **c** At 1 dpf, the neural tube and a head *Anlage* have formed (stage 10 shown). **d**, **e** By 2 dpf (stage 14), the embryos have developed pigmentation on the yolk, and a clear head can be distinguished, with presence of the mid-hindbrain boundary, otic vesicles and unpigmented eyes. At this stage, embryos are still surrounded by their chorion and the embryo shown has been manually dechorionated. **f**, **g** By 3 dpf (stage 15), the anterior-posterior axis has elongated further, pectoral fin *Anlagen* have appeared, and a clearly beating heart is visible. The eyes have developed light pigmentation. Also at this stage, embryos are still surrounded by their chorion, and the embryo shown has been manually dechorionated. **h**, **i** By 4 dpf (stage 17), the head has lifted up from the yolk, strong blood circulation through the heart is visible, and extensive vasculature runs across the yolk. All stages are indicated following the staging table for Nile tilapia [41]. Abbreviations: dpf: days post fertilization; st.: stage; BD: blastodisc; NT: neural tube; MHB: mid-hindbrain boundary; E: eye; OV: otic vesicle; PF: pectoral fin; H: heart; HA: Head *Anlage*

**Fig. 3:**
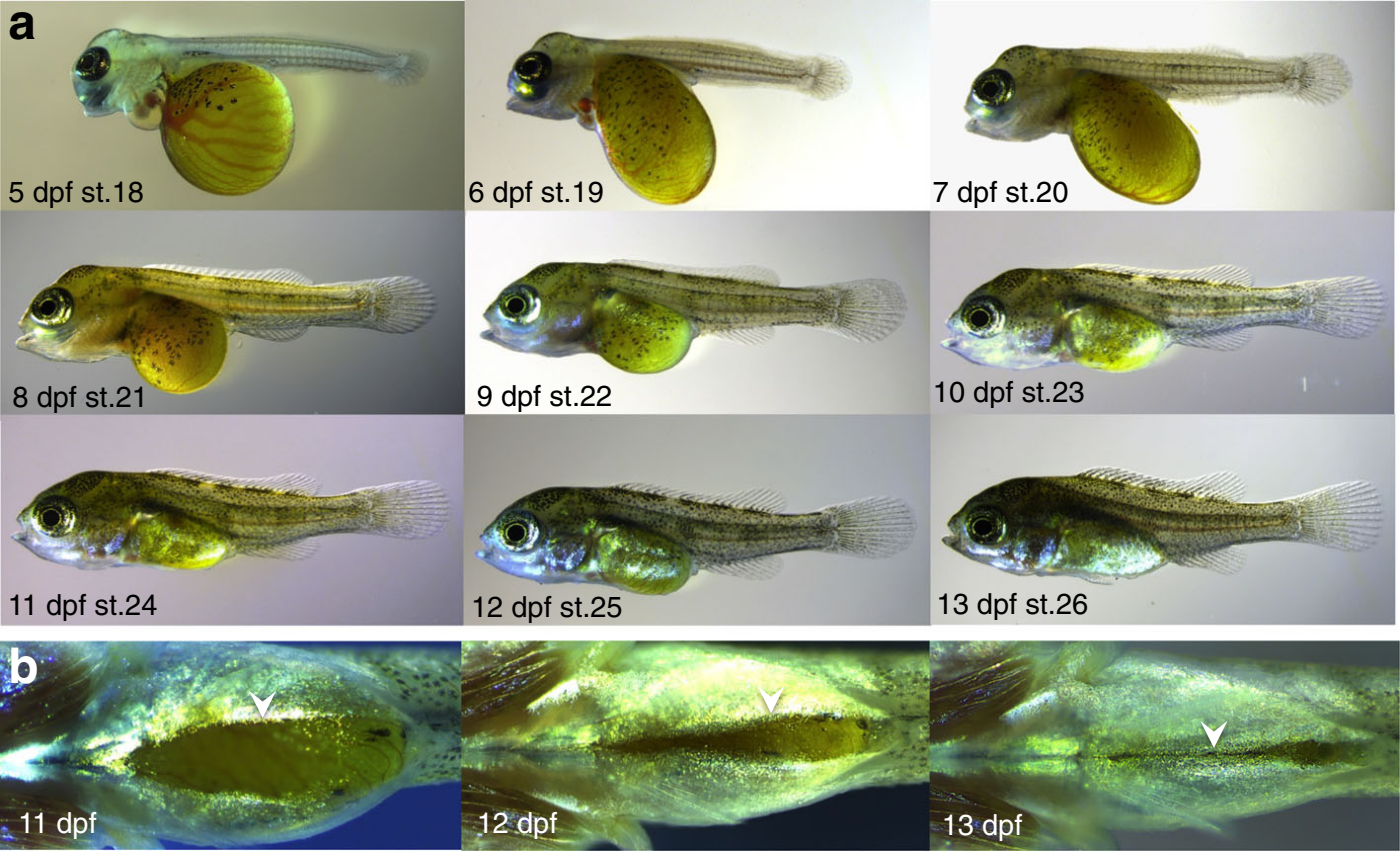
Late embryonic development of *A. burtoni*. **a** After hatching, embryos rapidly develop their adult morphology, whereby at 13 dpf essentially all structures of the body plan are present. **b** Ventral view during 11–13 dpf shows progressive closure of the body wall over the yolk during these stages (white arrowheads). All stages are indicated following the staging table for Nile tilapia [41]. Abbreviations: dpf: days post fertilization; st.: stage

Here, we give a brief overview of the embryonic development of *Astatotilapia burtoni*, which is very similar to the development of other *Haplochromine* mouth brooding cichlids, as for instance described before [40–42], but quite different from substrate spawning species [43]. The description given here aims to assist in determining the approximate age of clutches for which the date of fertilization is unknown, rather than providing an exhaustive staging table. In general, we found the widely used cichlid staging system, as established for Nile tilapia [41], to be useful for *A. burtoni*, which has a very similar speed of development. In Figs. 2 and 3 the corresponding stages from the Nile tilapia staging system are indicated. As however the numbering of these stages increases after hatching with one stage per day [41], and we describe the progression of some skeletal elements at a higher temporal resolution, we use hours or days post fertilization (hpf and dpf, respectively) to indicate the age of the embryos in the description of the skeletal ontogeny.

Eggs and early embryos of *A. burtoni* typically have an oblong shape and tend to rest on their side (which is different from zebrafish or *Xenopus* eggs, which usually have the animal pole oriented upwards). The blastodisc is located on the animal pole of the yolk (blastula shown Fig. 2a, stage 5). In contrast to zebrafish or medaka, the yolk is not transparent but opaque yellow, which at early stages (fertilization day to 1 dpf) can make observations on the embryonic *Anlagen* difficult. On the day of fertilization, the embryos pass through blastula stages and enter gastrulation. Gastrulation is completed by the second day, and the anterior-posterior axis can be distinguished by the formed neural tube (Fig. 2b, c, stage 10). Anteriorly, a thickening of the neural tube indicates the forming head (Fig. 2c). At these early stages, the chorionic membrane surrounds the embryos, and dechorionation is difficult without puncturing the yolk. Damaged embryos will usually not survive and will dissociate rapidly. If dechorionated embryos are required at these stages (for instance for in situ hybridization), it is usually more practical to dechorionate them after fixation, or alternatively, to dechorionate and immediately fix the embryos.

At 2 dpf, embryos (Fig. 2d, e, stage 14) have a clearly formed head with unpigmented eyes, otic vesicles, and the isthmus of the midbrain-hindbrain boundary is apparent. At 3 dpf, head development has progressed, and eyes have developed light pigmentation. (Fig. 2f, g, stage 15). A beating heart with relatively little blood circulation can be observed, as well as pectoral fin buds (Fig. 2g). At 4 dpf (Fig. 2h, i, stage 17), pigmentation in the eyes has increased, and the head has lifted upwards from the yolk. The heart is well developed with strong blood circulation, and extensive vasculature is running across the yolk. At 4 dpf, embryos hatch from their chorionic membrane, which can often be induced by gentle swirling of the petri dishes or pipetting up and down of the embryos.

After hatching (Fig. 3a, stage 18), embryos remain oriented on their sides until 7 dpf when they will right themselves and start swimming actively. Although many aspects of the body plan are apparent at this stage, we prefer to refer to them as “embryos” instead of “larvae”, according to the definitions used by Ballon 1999 [19] (and see discussion), since they do not actively feed yet. Between day 7 and day 13 (stage 20 – stage 26), embryonic development is completed, and the body wall has closed over the gut (Fig. 3b).

After 2 weeks of development, the juvenile fish have attained the adult body plan and start feeding. This is also approximately the time when juveniles would first venture out of the mother’s mouth during natural incubation [32]. In comparison to indirect-developing species, such as zebrafish [22] and medaka [23], development in *A. burtoni* stands out by its rapid development of the adult morphology, without the interlude of a prolonged, free-living and feeding larval stage.

### Osteology and axial meristics of the *A. burtoni* post-cranial body plan

**Fig. 4:**
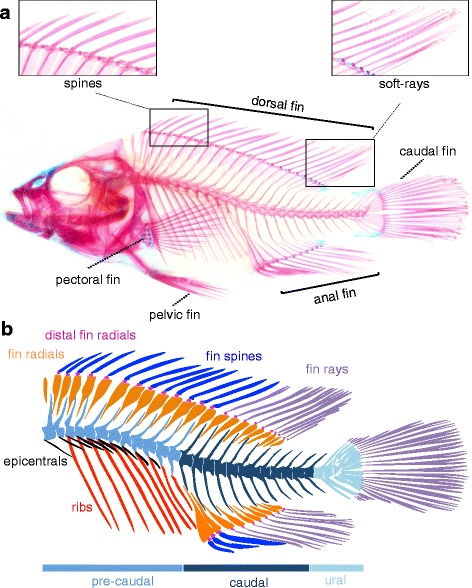
Osteology of the axial and median fin skeleton of *A. burtoni.* **a**, **b** Alizarin red/Alcian blue stained and cleared skeleton of an adult fish shows a typical teleost skeleton, consisting of a spinal column with associated elements, and pectoral, pelvic, dorsal, anal and caudal fins. The axial skeleton consists of pre-caudal, caudal and ural regions. Vertebral centra and associated arches and spines are drawn as fused units. Two anterior ribless vertebrae followed by around 10 rib-bearing vertebrae form the pre-caudal region. The caudal region consists of around 12 ribless vertebrae. The vertebral column terminates posteriorly in the ural region, which consists of highly modified vertebral elements and arches supporting the caudal fin rays. Other accessory elements present to the vertebrae are the epicentrals, also known as dorsal ribs (see main text), which are membrane bones without homologs in tetrapods. In panel **a** note the presence of the pelvic fins on the anterior abdomen at the same anterior-posterior level as he pectoral fins. Dorsal and anal fins consist of an anterior domain containing fin spines and a posterior domain with soft fin rays, each shown for the dorsal fin in a zoom-box in panel **a**

The post-cranial skeleton of *A. burtoni* shows a typical teleost organization with presence of median fins (anal, dorsal, caudal), paired fins (pectoral and pelvic) [24, 25, 44–47], a urophore complex, which supports the caudal fin rays [24, 44, 48–50], and an axial skeleton, which in addition to vertebrae [51, 52], contains dermal bones [53] absent from tetrapods (Fig. 4a,b).

The axial skeleton of *A. burtoni* consists of 27 (*N* = 2) or 28 (*N* = 7) vertebrae including the urostyle, the terminal vertebral element that forms part of the caudal fin urophore complex. The axial skeleton can be divided into pre-caudal, caudal and ural regions. The pre-caudal region consists of two anterior ribless vertebrae (*N* = 9), followed by 11 (N = 2) or 10 (N = 7) rib-bearing vertebrae. In addition to the ribs, additional rib-like dermal bones are present. These elements have been commonly referred to as “dorsal ribs”, and their homology to ribs in non-teleosts has been intensely disputed (see Britz and Barsch [54]). The current view is that these elements are teleost specific intermuscular bones that form in the myoseptum between epaxial and hypaxial musculatures, and have no homology to the ribs present in basal actinopterygians, sarcopterygian fishes or tetrapods [53, 54]. Here, we will refer to these structures as epicentrals following Patterson and Johnson 1995 [53]. The epicentrals are present from the 1st till the 9th (*N* = 1), 10th (N = 7) or 11th (N = 1) vertebra, and project laterally from the centra at a more dorsal position than the true ribs. Neural arches, which envelope the spinal cord, are present on all vertebrae except the urostyle. Haemal arches, which envelope and protect vasculature, define the start of the caudal region, and their occurrence is always mutually exclusive with the presence of ribs (*N* = 9). The vertebral column ends in the urophore complex, here defined following Monod, 1968 [50], as the vertebral elements that directly support the caudal fin rays, which consists in *A. burtoni* of two vertebrae plus the urostyle (see the paragraph on the caudal fin below).

The dorsal and anal fins are supported proximally by endochondral radials that are lodged internally between the neural and haemal spines (which are formed by the distal extensions of the neural and haemal arches). The radials articulate through the distal radials with the dermal fin rays, which form the external support of the fins. We prefer not to use the common ichthyological term “pterygiophore” here, which can refer to radials as well as rays in the anal and dorsal fins. Therefore, this indication is not informative for the embryonic origin of these elements, nor for the homology relationships between the supports of the median and paired fins, for the latter of which the expression pterygiophore is in general not used.

In cichlids, the dorsal and anal fins consist of two different classes of fin rays, namely spines anteriorly and soft-rays posteriorly (Fig. 4 a, b). Spines differ from soft-rays by stronger ossification, lack of segmentation, and ending in a sharp point instead of bifurcating [55, 56]. Fin spines have evolved as a defense mechanism against gape-limited predators [57], and are considered a hallmark innovation for acanthomorph fish [55, 58], the relatively young but very successful radiation of teleosts to which cichlids belong (although it needs to be noted that fin spines have evolved multiple times independently in lineages such as carps, catfish and sturgeons [56]). The dorsal fin of *A. burtoni* has 13 (*N* = 2) or 14 (*N* = 7) spines, and 9 (*N* = 3) or 10 (*N* = 6) soft-rays. In the anal fin there are 3 (*N* = 9) spines and 9 (N = 7) or 8 (N = 2) soft-rays. The osteology of pectoral, pelvic and caudal fin complexes are discussed in more detail in their respective sections (Figs 7, 9, 10 respectively).

### Development of the axial skeleton - vertebrae, arches and ribs

The vertebral column of vertebrates consists of two features: i) vertebral centers, which form around an embryonal notochord and form the central axis of the trunk skeleton, and ii) the haemal and neural arches, which attach to the centra ventrally and dorsally, and envelope the axial blood vessels and the spinal cord [51]. Although in the adult skeleton, centra and arches are seamlessly fused into a single morphological unit, these structures have distinct developmental and evolutionary trajectories [51, 59]. The accessory elements to the spinal column in the pre-caudal region considered here are the ribs and the epicentrals [53, 54].

**Fig. 5:**
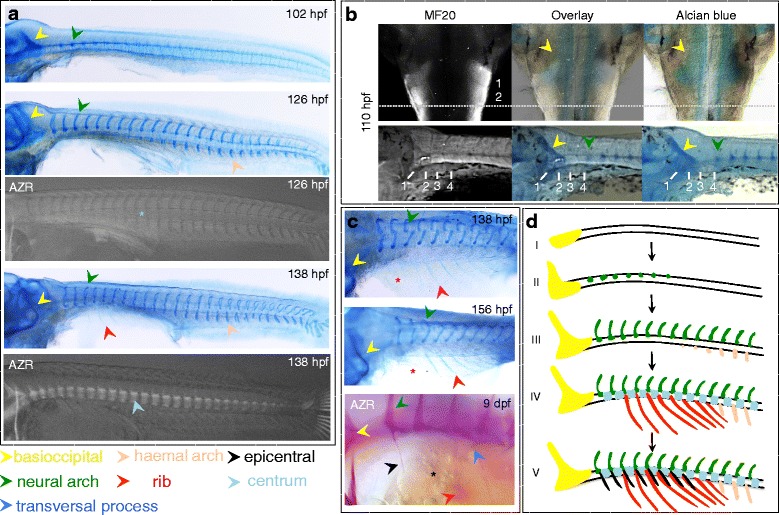
Development of the axial skeleton of *A. burtoni.* **a** Neural and haemal arches develop between 102 hpf and 126 hpf, as shown by Alcian blue staining. In teleost fish, vertebral centra do not form through a cartilaginous intermediate, and ossified centra become first visible by Alizarin red staining (fluorescent microscopy image) at 138 hpf. Blue asterisk in 126 hpf indicates absence of mineralized centra. **b** Visualization of somites, using the muscle sarcomere specific antibody MF20 together with Alcian blue staining, shows that the basioccipital (yellow arrowhead) has formed by 100 hpf within somites 1 and 2, and extends up to the somite 2–3 boundary. The first developing neural arch is present in somite 4. This shows that, as in other anamniotes, the basioccipital develops from the first three somites. The horizontal dotted line indicates the somite 2–3 boundary. **c** Ribs develop around 138 hpf (red arrowhead). Interestingly, we observe what appear to be rib *Anlagen* associated with pre-caudal vertebrae 1 and 2 (indicated with a red asterisk), which in the adult skeleton are not rib-bearing. At 156 hpf, these “cryptic” ribs are however no longer present (indicated with a red asterisk). Epicentrals develop much later and only start developing around 9 dpf as membrane bones (black arrowhead indicates epicentral on pre-caudal vertebra 1; the black asterisk indicates the forming epicentral on pre-caudal vertebra 2; the blue arrowhead indicates a transversal process on pre-caudal vertebra 4, which is not further discussed here). **d** The axial skeleton forms in the following sequence: the first element to appear is the basioccipital (stage I, before 100 hpf), followed by the formation of neural and haemal arches dorsal and ventral of the notochord (stage II and III). Further elements to appear are the vertebral centra and the ribs (stage IV), followed by the epicentrals (stage V). Abbreviations: dpf: days post fertilization; hpf: hours post fertilization; AZR: Alizarin red

In *A. burtoni,* the first vertebral elements to form are the chondral condensations of the neural and haemal arches, which appear as paired structures around 100 hpf and develop in an anterior to posterior sequence (Fig. 5a and stage II, III, Fig. 5d). The vertebral centra start forming later, and form through intramembranous ossification without a cartilaginous intermediate, and are first detected around 138 hpf using Alizarin red staining (Fig. 5a and stage IV, Fig. 5d). The ribs become visible as cartilaginous elements around 138 hpf (Fig. 5c and stage IV, Fig. 5d). In the adult skeleton, the most anterior ribs are present on the third vertebra. However, around the first time of appearance we also do observe presumptive rib *Anlagen* staining with Alcian blue at the two more anterior somites (Fig. 5c). The development of these cryptic rib *Anlagen* does however not continue, and these have disappeared by 156 hpf. The epicentral bones form much later during development, and form as intramuscular bones in anterior to posterior sequence, with the first bone of this series arising on the first vertebra (Fig. 5c and stage V, Fig. 5d) (shown at 9 dpf), and with a subsequent appearance in anterior-posterior order along the pre-caudal region of the trunk.

In vertebrates, somites not only contribute to the postcranial skeleton, but also to the formation of the posterior skull. In amniotes, the occipital somites are the anterior-most five somites, which form part of the skull base [60–62], while in anamniotes (axolotl and zebrafish), only the first three somites contribute to the occipital arch [52, 63, 64]. We used immuno-staining with the muscle sarcomere specific antibody MF20 [65] in combination with Alcian blue staining to visualize the correspondence between the basioccipital and the somites in *A. burtoni*. The first basioccipital condensations, which form around 100 hpf, are visible in somite 1, and by 110 hpf extend up to the somite 2–3 border, while the neural arch condensations of the first vertebra develop inside somite 4 (Fig. 5b). This pattern is consistent with the anamniote pattern, whereby the first three somites contribute to the occiput. In actinopterygian fishes, the ancestral condition, as present in basal non-teleosts (e.g. *Amia calva* and gar (*Lepisosteus* sp.)) [66], is the fusion of one or more vertebral elements with the posterior skull. As has been previously reported for other teleost species, we do not observe any contribution of pre-formed vertebral elements to the skull. Therefore, the occipital-trunk transition in *A. burtoni* conforms to the generalized teleostean condition as proposed by Patterson and Johnson [53]. Namely, it consists of three myotomal segments associating with the skull, which as in other anamniotes, derives from the first three occipital somites, followed by two ribless vertebrae, and the first rib is present on the third vertebrae.

### Development of the dorsal and anal fins

**Fig. 6:**
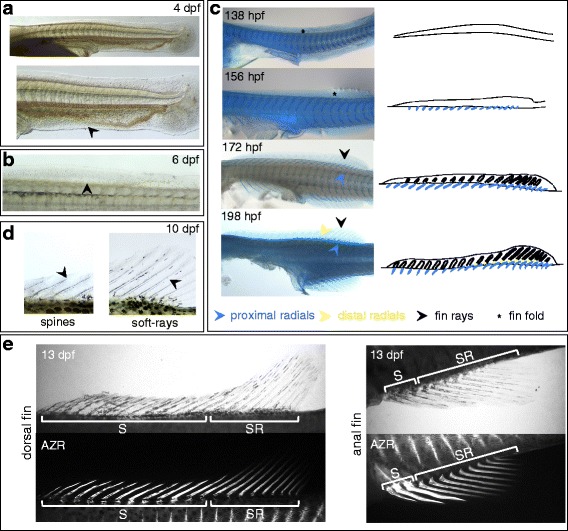
Development of the anal and dorsal fins of *A. burtoni*. **a** The first dorsal and anal fin *Anlagen* are visible at hatching day (4 dpf) as a fin fold that is continuous with the caudal fin. In the anal fin fold, the domain of the final anal fin is recognizable by the lack of vascularization (black arrowhead in lower panel), which is restricted to the part of the fin fold that will degenerate. **b** At 6 dpf, mesenchymal condensations become visible proximally in the posterior dorsal and anal fins (indicated with a black arrowhead for the dorsal fin). These condensations correspond to tissue surrounding the forming fin radials (see transition from 138 hpf to 156 hpf in panel **c**). **c** Alcian blue staining shows the progression of dorsal and anal fin formation. At 138 hpf, a fin fold is present without detectable skeletal structures (asterisk). By 156 hpf, fin radials have formed (blue arrowhead), but no ray structures are apparent yet in the fin fold (asterisk). By 172 hpf, fin rays start forming (black arrowhead). The last elements to appear are the distal radials in between the proximal radials and the fin rays, and these appear around 198 hpf. **d** Initially, fin spines and soft-rays look very similar. By 10 dpf, their differentiation has progressed and clearly shows diverging developmental trajectories. Fin spines have developed a pointy ending, and segmentation is apparent in the soft-rays (black arrowheads). **e** At 13 dpf, ossification in the soft-rays and spines in dorsal and anal fins has progressed, and clearly demonstrates stronger ossification of the spines compared to soft rays. Upper panel shows bright-field view, lower panel shows fluorescent imaging for Alizarin red staining in the same specimen. Abbreviations: dpf: days post fertilization; hpf: hours post fertilization; AZR: Alizarin red; S: spines, SR: soft-rays

Dorsal and anal fins consist of proximal radials, located inside the body, which articulate with the external dermal fin support, the fin rays, through an articulation consisting of the distal radials (see above and Fig. 4a, b). Dorsal and anal fins develop synchronously and their development is therefore described here together. The first *Anlagen* for dorsal and anal fins are present at hatching day as a fin fold that is continuous with the caudal fin (Fig. 6a). This fin fold will, however, only differentiate into an adult fin in the anterior part, and degenerate in between the anal/dorsal fins and the caudal fin, consistent with the situation in zebrafish, where it has been shown, using lineage tracing, that early mesenchyme in the dorsal and anal fins does not contribute to the adult skeleton [67]. At these early stages, most of the anal fin fold is strongly vascularized. The anterior absence of vasculature, however, marks the prospective domain of the final anal fin (Fig. 6a, arrowhead lower panel). At 6 dpf, mesenchymal condensations can be observed in the dorsal and anal fins (Fig. 6b arrowhead). Alcian blue staining at 156 hpf (Fig. 6c) shows that these condensations correspond to mesenchyme surrounding the endochondral radials, which at posterior positions extend beyond the trunk perimeter, while at more anterior positions they are less well visible as they develop entirely within the body. At 172 hpf, fin rays have started forming in dorsal and anal fins. Although fin rays are dermal bone and do not form through a cartilaginous precursor, they do stain slightly using Alcian blue, an observation we also made for some other dermal skeletal elements, such as the cleithrum (see below). Ray condensations form at the same time in the spiny and the soft-rayed domains of the fin. Initially, spines and soft-rays look very similar, but by 10 dpf they have clearly adopted different morphological identities, and the spiny endings on spines and segmentation of soft-rays are present (Fig. 6d). By 13 dpf, differential ossification of soft-rays and spines in dorsal and anal fins is detected using Alizarin red staining, and the heavier ossified character of fin spines has become apparent. Distal fin radials have formed by 198 hpf in the central part of the soft-ray domain of dorsal and anal fins, and will also appear slightly later throughout the more anterior and posterior parts of the fins (Fig. 6c).

Not only do dorsal and anal fins develop virtually synchronously, also the anterior spiny part and the posterior soft-rayed part develop largely simultaneously. Only the middle soft-ray domain is slightly ahead of the more posterior soft-ray and the anterior spine domains. Fin spines are considered an evolutionary invention associated with the rise of acanthomorph (spiny-rayed) fishes [58], and it has been argued that the spiny and soft-ray domains of the dorsal fin comprise distinct developmental and evolutionary modules [68]. Their developmental sequence in *A. burtoni* strongly suggests that they are produced under influence of the same developmental signals and that they acquire their different identity only later through subsequent patterning as typical for serially homologous elements [69, 70].

### Development of the pectoral fins and girdles

**Fig. 7:**
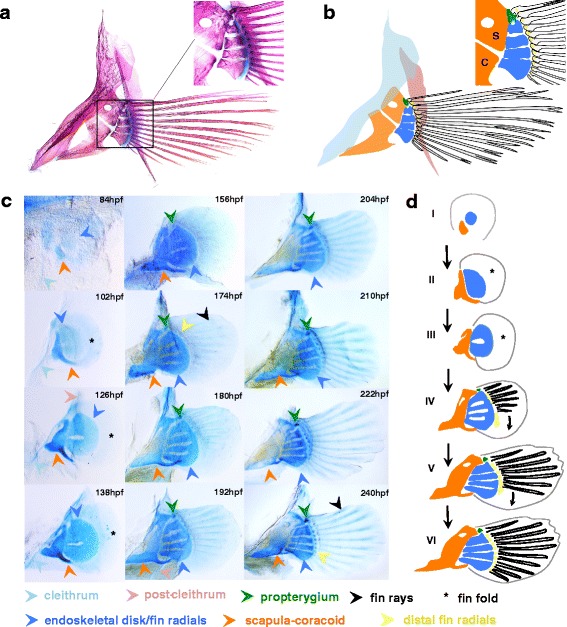
Development of the pectoral fins and girdles of *A. burtoni*. **a**, **b** The pectoral girdles and fins of *A. burtoni* show the typical teleost anatomy. The girdle consists of two dermal bones, the cleithrum and the post-cleithrum, and two endochondral bones, the scapula and coracoid (indicated S and C in panel **b**). The pectoral fins consist of proximal radials that articulate via distal radials with the main external fin support, the dermal fin rays. The propterygium (see main text) is present as a separate radial during embryogenesis but is in the adult fused to the scapula. Shown is an Alizarin red/Alcian blue stained pectoral fin/girdle complex of an adult fish. **c**, **d** Alcian blue stained sequence of pectoral fin development (see main text for details). The cleithrum is indicated from 84 hpf to 138 hpf. Distal radials appear first at 174 hpf, and are present but not indicated in 180 hpf to 222 hpf. The post-cleithrum, which is only visible through its different contrast, is only indicated in 126 hpf and 192 hpf. Abbreviations: hpf: hours post fertilization; S: scapula; C: coracoid

The osteology of the pectoral fins of *A. burtoni* adheres to a typical teleost structure, consisting of proximal radials that articulate through distal radials with dermal fin rays (Fig. 7a, b). Following the framework for comparative anatomy of the paired appendages as proposed by Carl Gegenbauer [71], the endochondral parts of the pectoral fin consist of a propterygium and four mesopterygial radials, while a metapterygium is absent [25, 72–74]. Four mesopterygial fin radials are present in adult fish, and the propterygium is fused to the scapula, although during embryonic development it is present as a distinct structure (see below). The shoulder girdle consists of two endochondral bones, the scapula and the coracoid that derive from a single chondral plate. In addition, two dermal bones are present, namely the cleithrum, which lies antero-lateral of the scapula-coracoid, and the post-cleithrum, located postero-medial of the fin [45, 75]. The small nodular bones of the distal radials provide the articulation between the fin rays and the proximal radials.

During the earliest stages of fin development (84–102 hpf, Fig. 7c and stage I and II, Fig. 7d), the pectoral fin and girdle are visible as two separate cartilaginous *Anlagen* for the scapula-coracoid and the fin radials, respectively. The cartilaginous *Anlage* for the radials is generally referred to as endoskeletal disk. In *A. burtoni* this structure is very transient (84–102 hpf, Fig. 7c and stage I, II, Fig. 7d), in contrast to its prolonged persistence in indirect developing teleosts, such as zebrafish, where it remains present during larval stages and does not segment into the radials until the transition into adults [25] (and see discussion). Although the cleithrum is a dermally ossifying bone [55], we find that it stains weakly using Alcian blue before its ossification, and can be seen lying rostral from the other girdle and fin elements (indicated in 84–138 hpf, Fig. 7c). In the Alcian blue stained series, the post-cleithrum is difficult to detect but is visible through its differential contrast, and can be seen behind the endoskeletal disk and radials (indicated in 126 and 192 hpf, Fig. 7c).

Sequential segmentation of the endoskeletal disk gives rise to the mesopterygial radials. The first segmentation, which partially separates the domain of the two anterior radials from the domain of the two posterior radials, occurs around 126 hpf (Fig. 7c and stage III, Fig. 7d). This is followed by the next segmentation anteriorly and posteriorly at around 156 hpf (Fig. 7c and stage IV, Fig. 7d). Initially, the radials stay joined at their distal edge, and segmentation progresses in anterior to posterior direction reaching full segmentation at 240 hpf (Fig. 7c and stage VI, Fig. 7d). The propterygium becomes visible at the anterior edge of the partially segmented endoskeletal disk at 156 hpf (Fig. 7c and stage IV, Fig. 7d), although it is not clear whether its formation is due to a de novo cartilage condensation, or whether it segments off the endoskeletal disk. The first distal radials become visible around 174 hpf, form in an anterior to posterior sequence, and reach the position of the most posterior proximal radial at 240 hpf (and subsequently their development continues more posteriorly). As is the case for the propterygium, we have not been able to determine whether the distal radials form through de novo condensations or by splitting from the fringe of the preformed cartilaginous elements of the radials.

The most distal elements of the fins, the dermal fin rays, do not form via cartilage precursors, yet stain lightly using Alcian blue (as is the case for the cleithrum and the dorsal and anal fin rays). In teleosts it is now well established that the fin rays, like the radials, are derived from mesoderm [76, 77], and they form from mesenchyme within an epidermal sheet, the fin fold [78] (indicated in 102 to 138 hpf, Fig. 7c and stage I, II, Fig. 7d). The first fin rays become visible around 156 hpf (Fig. 7c and stage IV, Fig. 7d), and appear in anterior to posterior sequence (as reported in ref. [79]) until stage 240 hpf (Fig. 7c and stage VI, Fig. 7d), when they have formed along the full anterior-posterior margin of the fin.

**Fig. 8:**
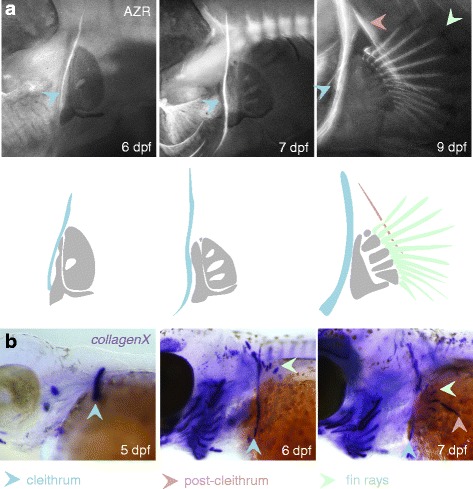
Development of the pectoral girdle of *A. burtoni*. **a** Visualization of ossification by fluorescent imaging of Alizarin red stained embryos. At 6 dpf and 7 dpf, only the cleithrum has ossified, followed by the fin rays and the post-cleithrum at 9 dpf. Embryos shown were also stained for Alcian blue, which in the fluorescent images shows cartilaginous elements as a dark counterstain. In the drawing these unossified endoskeletal girdle and fin elements are indicated in grey. **Cb** In situ hybridization for *collagenX* visualizes the ossifying dermal bones in the same sequence but approximately one day in advance of the mineralization process as detected by Alizarin red staining. Abbreviations: dpf: days post fertilization; AZR: Alizarin red

Because of the dermal composition of the pectoral girdle, we have further analysed its ossification pattern using Alizarin red staining (which visualizes mineralized bone) and in situ hybridization for the ossification marker *collagenX* (Fig. 8). The first bone in the pectoral fins and girdles to ossify is the cleithrum at 6 dpf, only followed at around 9 dpf by the post-cleithrum and the fin rays (Fig. 8a). In situ hybridization for *collagenX* shows that the expression of this gene foreshadows the bone mineralization process, and visualizes the shoulder girdle elements approximately 1 day earlier (Fig. 8b).

### Development of the pelvic fins and girdles

**Fig. 9:**
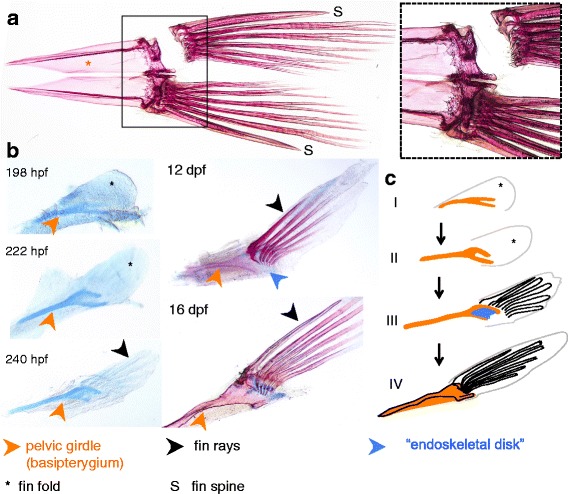
Development of the pelvic fins and girdle of *A. burtoni*. **a** Alizarin red/Alcian blue stain of the adult paired pelvic girdle (ventral view, both left and right side elements are present). The pelvic girdle consists of a single element (the basipterygium) that directly articulates with the fin rays, without presence of proximal or distal radials (see zoom in on the dissected left fin). The most anterior fin ray is unbranched and unsegmented, and has the identity of a spine (indicated with “S”), while the other rays have soft-ray morphology. **b**, **c** Alcian blue (198 hpf-240 hpf) and Alcian blue/Alizarin red stained (12 dpf − 16 dpf) pelvic girdle/fin complexes. The first elements of the pelvic fin skeleton appear around 198 hpf as a split chondrogenic condensation, which at 240 hpf has elongated and developed a distinct “forked” appearance. Between 240 hpf (10 dpf) and 12 dpf, the forked ends become connected through intermediate occurring cartilaginous growth, possibly homologous to the endoskeletal elements of the pectoral fins (blue arrowhead, indicated “endoskeletal disk”). Fin rays develop from 240 hpf onwards (indicated black arrowhead). Abbreviations: dpf: days post fertilization; hpf: hours post fertilization; S: spine

Contrary to the pectoral fins, the pelvic fins play a relatively subordinate role in fish locomotion, and have been frequently lost or become highly modified during evolution [80–82]. In the acanthomorph (spiny-rayed) fish, such as cichlids, the position of the pelvic girdle is located anteriorly below the pectoral girdle (in contrast to its ancestral position near the anus) [55, 83, 84] (see also Fig. 4a). During ontogeny, however, the pelvic appendages still arise at a conserved position around the anus, and migrate anteriorly to their final location after protrusion of the fin buds [84]. During development, pelvic fins appear at a much later stage than pectoral fins. In cichlids, the two pelvic fins and girdles are located side by side on the ventral midline. In contrast to the pectoral fins, the pelvic fin complex includes only a single girdle element (often referred to as basipterygium [73, 83]), which directly articulates with the fin rays, and, unlike in zebrafish [25], there are no free radial elements in *A. burtoni*. The most anterior fin ray has the identity of a spine, and is unsegmented and unbranched (Fig. 9a).

The first pelvic elements appear between 180 and 198 hpf (Fig. 9b and stage I, II, Fig. 9c), and are visible as a forked chondrogenic condensation corresponding to the future girdle. Between 10 and 12 dpf (240 hpf- 12 dpf, Fig. 9b and stage III, Fig. 9c), a cartilaginous condensation forms between the two girdle protrusions, and fills up the space between them. At this stage, there is a striking resemblance between the ontogenic trajectory of the pectoral and pelvic fin. In both, a forked girdle element is present with a cartilaginous plate in between. In the pelvic fins, this cartilage will however not continue to produce individual radial elements, but fuses with the girdle. By 10 dpf (240 hpf, Fig. 9b and stage III, IV, Fig. 9c), fin rays have appeared, and these articulate directly with the fused girdle-disk.

In adult teleosts, the pectoral and pelvic appendages and girdles are very different in their appearance. Also in more basal fish species, there exist significant differences between pectoral and pelvic appendages, contributing to the discussion considering their serial homology [85]. During the development of the pelvic and pectoral fins and girdles in *A. burtoni,* we noticed a hitherto, to our knowledge, undescribed pattern of similarity between the development of pelvic and pectoral appendages, whereby both first appear as a forked chondrogenic condensations corresponding to the girdle elements, followed by the formation of the endochondral disk in between the two distal girdle extensions. Perhaps as a result of the staging series used, this pattern has not been described in other teleosts, such as zebrafish [25] and sea bream [73]. Although this pattern may reflect a homologous as well as an analogous developmental trajectory, it nevertheless provides a striking resemblance between these appendages, and could represent a basic ontogenetic trajectory underlying the formation of both pectoral and pelvic girdles and appendages, worthy of further studies.

### Development of the caudal fin

**Fig. 10:**
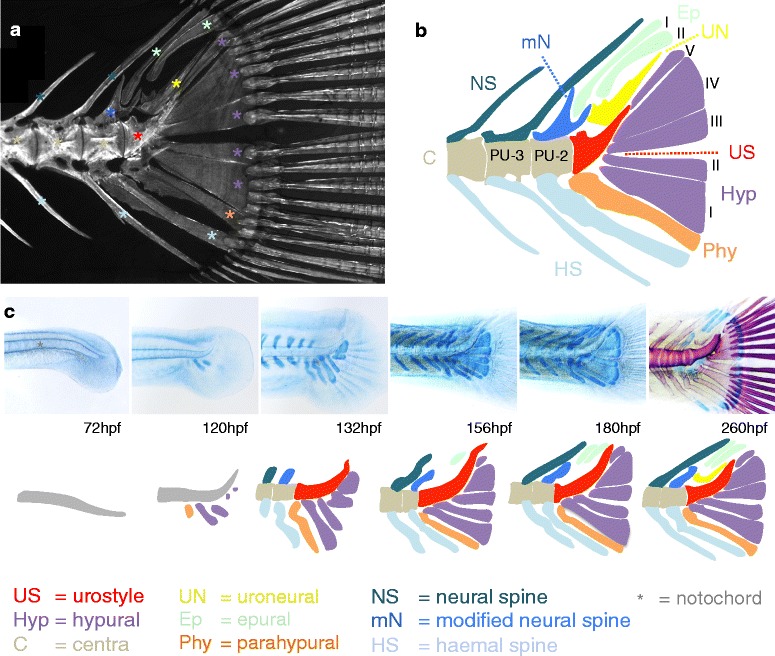
Development of the caudal fin of *A. burtoni*. **a**, **b** Osteology of the adult caudal fin of *A. burtoni* as detected by Alizarin red staining (fluorescence image in panel **a**. *A. burtoni* has a typical acanthomorph caudal fin consisting of five hypurals (I-V), a parahypural, two epurals (I, II), a urostyle, and two haemal and neural spines, contributing to the support of the dermal fin rays. Note the highly modified neural spine on the second pre-ural centrum (PU-2) that forms a complex articulation with the first epural. **c** Alcian blue staining shows that the first elements to appear are the hypurals and parahypural aroud 120 hpf. This is followed by the appearance of the haemal and neural arches on pre-ural centra 2 and 3 around 132 hpf. The epurals appear later, between 156 hpf and 180 hpf. By 260 hpf (Alcian blue/Alizarin red double stain), the uroneural has developed as a directly ossifying membrane bone. The developing notochord is indicated using a grey asterisk in the 72 hpf specimen. Abbreviations (except those  explained at the bottom of the figure): hpf: hours post fertilization

The homocercal caudal tail fin is considered a teleost evolutionary innovation that improves on the ancestral heterocercal fin, as for instance found in sturgeons and sharks, and has significantly contributed to the evolutionary success of teleosts [46, 66]. Where the ancestral heterocercal caudal fin is present along the ventral midline, the homocercal caudal fin faces backward with a strong external dorso-ventral symmetry, which combined leads to an increased propulsive capacity [86]. In its internal anatomy and ontogeny, the homocercal fin, however, still clearly shows its ventral evolutionary origin [66]. In contrast to the other median and paired fins, the fin rays in the caudal fin do not articulate with true radial-distal radial complexes, but instead are connected to highly derived haemal and neural spines (the hypurals, parahypural and epurals, respectively), as well as more anteriorly, to a single unmodified haemal and neural spine [24, 49, 87] (annotated *A. burtoni* adult caudal fin skeleton shown in Fig. 10a, b). The vertebral column terminates in the caudal fin complex with a highly derived vertebral element, referred to as the urostyle, which in cichlids consists of three fused vertebral centra [49, 87]. The two free vertebral centra anterior of the urostyle, which contain neural and haemal elements forming part of the caudal fin complex, are referred to as pre-ural vertebrae 2 and 3 [49, 50]. Additional elements present are the uroneural, which represents the derived fused neural arches of the urostyle, and a highly modified neural arch on pre-ural vertebra 2, articulating with the first epural. Together, these elements are generally referred to as the urophore complex [50].

The first elements to appear in *A. burtoni*, at approximately 100–120 hpf, are the chondrogenic condensations of the hypurals and parahypural, starting with hypural I (developmental sequence shown in Fig. 10c). The haemal and neural arches on pre-ural vertebrae 2 and 3 develop slightly later, around 132 hpf, in phase with the development of the haemal and neural arches of more anterior vertebrae. On the dorsal side of the urophore complex, this leaves a distinct gap at the position of the future epurals and uroneural. Also, during this developmental window (100–132 hpf), the notochord flexes strongly dorsally. Only at 156-180 hpf, the epurals and the terminal hypural V become visible as cartilaginous elements. The uroneural develops as a membrane bone without a cartilaginous intermediate and ossifies during subsequent stages of development (visible at 260 hpf – exact time point of ossification not determined). This pattern of development is highly similar to that described for other teleosts such as sea bream [88] and zebrafish [24, 48]. Interestingly, there appears to be a clear heterochronic shift in development of the derived hypurals/parahypural and epurals, and the flanking unmodified haemal and neural arch of the urophore complex. The unmodified haemal and neural arch form in concert with the other neural and haemal arches of the posterior axial skeleton, while most of the ventrally located hypurals/parahypural form earlier, and the dorsally located epurals form later. Therefore, the teleost urophore complex shows a temporal modularization between ventral and dorsal elements. We would like to suggest that this dorso-ventral heterochrony, with a precocious expansion of the ventral skeletal elements in combination with a delayed formation of dorsal elements, in fact might facilitate the dorsal flexion of the vertebral column, which is so characteristic of the homocercal caudal fin. Also, the formation of the uroneural, as a late ossifying membrane bone instead of as an endochondrally ossifying bone with a cartilage precursor (which is the default state for neural arches), appears compatible with this idea, as it could contribute to the process of dorsal flexion.

### Development of scales in *A. burtoni*

**Fig. 11:**
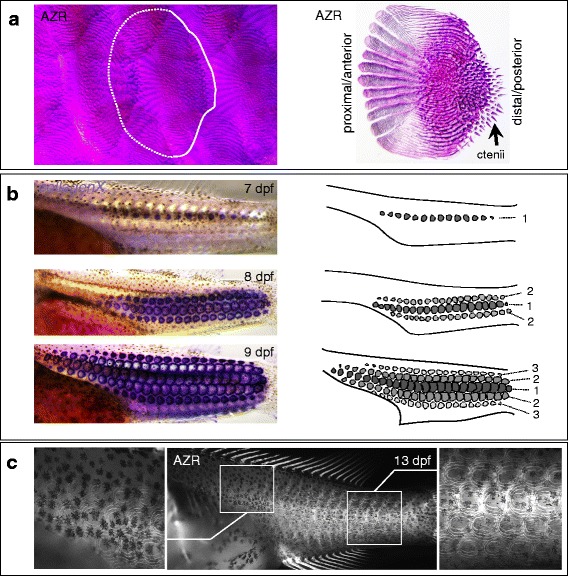
Scale development in *A. burtoni*. **a** Adult scales stained using Alizarin red. Scales are present in an organized arrangement, whereby their proximal-distal axis is aligned anterior-posteriorly along the trunk. Proximally, scales are overlapped by the distal part of their anterior/lateral neighboring scale(s). In the left panel, the outline of one scale is drawn using a dotted line for the proximal part that is covered by the flanking scales. In the right panel, a close up of a single scale is shown. The scales consist of concentric rings of bone, and spike elements distally, the ctenii. The scales attach to the body through their proximal end. **b** During development, scale *Anlagen* are first detected at 7 dpf, using in situ hybridization with the ossification marker *collagenX*, as a single row along the posterior midline (indicated “1” in the drawing to the right). At 8 dpf, one more row dorsally and one more row ventrally have appeared (indicated “2” in the drawing to the right), while the initial row has expanded anteriorly. At 9 dpf, this process has been repeated with the appearance of an additional dorsal and ventral row of scales (indicated “3” in the drawing to the right). **c** At 13 dpf, bone mineralization in the scales is present along the posterior trunk where they were first formed (zoom-in right panel), while more anteriorly, mineralization is still absent or less pronounced. Shown is a fluorescence image of an Alizarin red stained specimen. Abbreviations: AZR: Alizarin red; dpf: days post fertilization

In teleosts, the squamation pattern of the trunk is formed by an organized tiled arrangement of leptoid scales, whereby the proximo-distal axis of each scale is aligned anterior-posteriorly along the body axis. As a result, the proximal part of each scale (through which it is attached to the body) is covered by the distal part of its anterior neighbor(s), while its own distal part overlaps the proximal part of the posteriorly flanking scale(s) (Fig. 11a). The leptoid scales consist of concentric rings of bone referred to as annuli (Fig. 11a). Cichlids possess leptoid scales of the “ctenoid” type, which are characterized by the distal presence of spikes, the ctenii (Fig. 11a).

We assayed the developmental appearance of scales using Alizarin red staining and in situ hybridization using the ossification marker *collagenX*. The first scale *Anlagen* become visible on day 7 along the posterior midline (7 dpf, Fig. 11b). By day 8, this line of squamation has extended anteriorly, and two new rows of scales have appeared, one ventrally and one dorsally of the initial row (8 dpf, Fig. 11b). By day 9, the three rows have continued expanding anteriorly, while at the same time again two more rows of scales have appeared ventrally and dorsally (9 dpf, Fig. 11b). This pattern of scale appearance resembles that described for other fish, such as zebrafish [89] and other teleosts [90]. Ossification, or at least bone mineralization, as assayed by Alizarin red staining, occurs slightly later during development, and also proceeds in a posterior to anterior sequence, whereby at 13 dpf the scales along the posterior midline are mineralized, while more anteriorly mineralization is still absent (13 dpf, Fig. 11c).

### Development of the cranium in *A. burtoni*

The focus of our description of the developmental osteology of *A. burtoni* has been on the post-cranial skeleton, particularly since a very detailed description of the cranial ontogeny is already available for the closely related Nile tilapia [91]. For completeness, we include here a brief description of the development of the chondrified element of the head, and the subsequent ossification of the jaw and skull bones, as we have observed in *A. burtoni*.

**Fig. 12:**
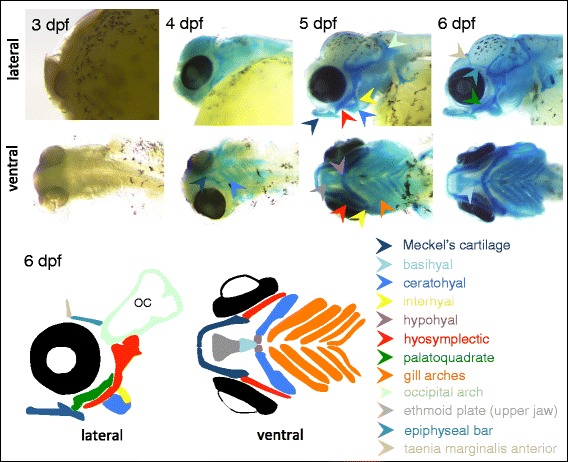
Chondrification of the cranial skeleton in *A. burtoni*. Chondrified elements of the cranio-facial skeleton are detected using staining with Alcian blue. At 3 dpf, no chondrogenic differentiation of the craniofacial skeleton is yet detected At 4 dpf, the first elements corresponding to the pharyngeal skeleton become vissible and differentiate rapidly, so that at 5 dpf, the full complement of pharyngeal cartilages has formed. The schematic illustration of the chondrogenic cartilages present is for 6 dpf. Abbreviations: dpf: days post fertilization

The first facial cartilages appear in the lower pharyngeal skeleton at 4 dpf, and include the gill arches, the ceratohyal, Meckel’s cartilage, the maxilla, and some dorsal elements of the upper jaw. At 5 dpf, cartilaginous derivatives from all seven pharyngeal arches are present in the lower pharyngeal skeleton, and have formed Meckel’s cartilage, the basihyal/hypohyal/ceratohyal/interhyal complex, and the five posteriorly located gill arches (5 dpf, 6 dpf, Fig. 12). In the upper jaw, the hyosymplectic, ethmoid plate and palatoquadrate have formed, the latter of which is more pronouncedat 6 dpf. Also at 6 dpf, additional antero-dorsal condensations appear in the dorsal skull.

**Fig. 13:**
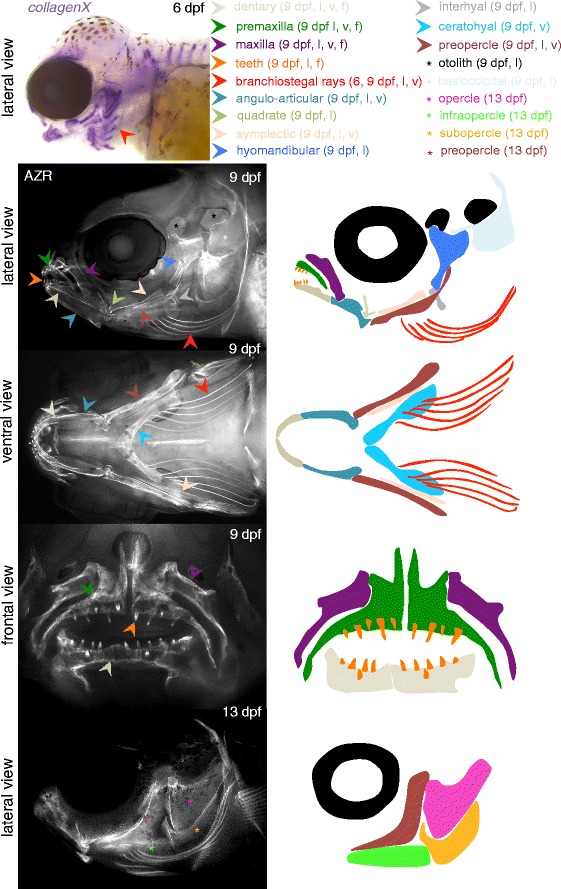
Ossification of the cranio-facial skeleton in *A. burtoni*. At 6 dpf, ongoing ossification can be detected in the craniofacial skeleton using in situ hybridization with *collagenX*. This clearly visualizes the formation of the branchiostegal rays (red arrowhead), which form without a cartilage intermediate (compare 6 dpf in Fig. 12). At 9 dpf, most of the adult bones of the cranio-facial skeleton have started ossification, as shown by Alizarin red staining (fluorescence microscopy images shown), and the formation of teeth has started (orange arrowhead). Full formation of the complete opercular series (opercle, infraopercle, subopercle and preopercle) is shown for a 13 dpf specimen. Note that many bones in the skull form without a cartilaginous intermediate. Therefore, there is no one to one correspondence between the chondrogenic elements in Fig. 12 and the ossifying elements in Fig. 13. The legend in the figure indicates for which specimen each anatomical element is indicated. Abbreviations: l: lateral view; v: ventral view; f: frontal view; dpf: days post fertilization

Analysis at 6 dpf for ongoing osteogenesis using in situ hybridization with *collagenX*, shows the ongoing ossification at various positions in the jaw and skull, most prominently, however, in the forming branchiostegal rays (6 dpf, Fig. 13). At 9 dpf, most of the facial bones have started ossification. It is important to note that many of these bones form by independent intramembraneous ossification events, and not through the endochondral ossification of the already present facial cartilages. Therefore, many of the facial bones are never present as a cartilaginous precursor, and many cartilages do not directly give rise to an adult facial bone. At 9 dpf (9 dpf, Fig. 13), clear ossification of the lower and upper jaw bones is present, and teeth have started to form Also the branchiostegal rays have ossified. Full ossification of the oppercular series is demonstrated in a 13 dfp specimen (13 dpf, Fig. 13).

## Discussion

### Direct- versus indirect-development; *A. burtoni* versus zebrafish

There has been considerable discussion over the definition of indirect- versus direct-development in fishes and the criteria for the delimitation of embryonal, larval and adult life-stages. Part of this is due to the different prioritization of phenomena such as hatching and the onset of feeding by different authors, which has lead to incompatible views e.g. [19, 92]. Here, we follow the synthesis provided in Ballon 1999 [19], and consider the embryo to larva transition as the point where an embryo becomes dependent on exogenous feeding, but only so if at this point the formation of the body plan has not been completed (i.e. the embryo has not yet become a “definitive phenotype” [19]). In many teleost species, the timing of the embryo to larva transition is related to the amount of yolk provided in the egg. Sparse yolk provision is related to an early transition to the larval stage, and these larvae generally possess many embryonal characters, and still lack essential aspects of the body plan [18, 19]. Embryos from species with large yolk-rich eggs, such as *Haplochromine* cichlids, become only dependent on feeding at a relatively later stage, when nearly all aspects of the body plan have developed (i.e. a “definitive phenotype”), making them in effect transform directly from a non-feeding embryo into a feeding juvenile fish, without the interlude of a larval stage [19].

**Fig. 14:**
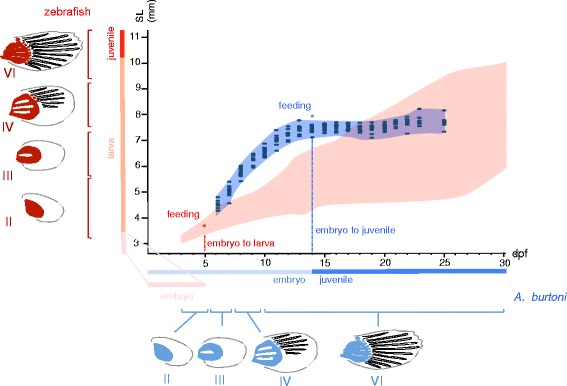
Direct- versus indirect-development – *A. burtoni* versus zebrafish. The direct-development of *A. burtoni* (blue) is characterized by the completion of the body plan before the onset of feeding (embryo to larva transition at 14 dpf). This is reflected in a very homogenous progression of development, as has been described in this paper, and a very homogenous overall growth with only minor variation in standard length (Y-axis, data points included for 10 embryos/juveniles per day coming from different clutches). In comparison, zebrafish (red) develops only synchronously until the start of the embryo to larva transition, marked by the onset of feeding at 5 dpf. After this point, growth trajectories of individual fish strongly diverge (data range included from Parichi et al. 2009 [22], a very similar range was described in Grandel & Schulte-Merker 1998 [25]). The zebrafish' larva- to juvenile transition is indicated after references [4, 22]. When we consider the development of the pectoral fins for zebrafish (after [25]) and *A. burtoni* (this article), we see that in both species these follow very similar developmental trajectories (stages indicated after Fig. 7). In *A. burtoni*, pectoral fin formation is completed before 10 dpf, well before feeding stages, and differentiation occurs at a steady progression, and is predictable amongst individuals from the same clutch (developmental progression indicated along the X-axis in relation to time after fertilization). In zebrafish, pectoral fin formation starts similar with the formation of the endoskeletal disk (stage II). From the embryo to larva transition onward however, overall development slows down and growth becomes heterogeneous amongst similarly aged larvae. Therefore, time is not a good predictor of the progression of pectoral fin development, but standard length has to be used instead (pectoral fin stages indicated along the Y-axis in relation to standard length). Abbreviations: dpf: days post fertilization; SL: standard length

Now, in species where there is a pronounced larval stage, embryonal characters, such as the endo-skeletal disk of the pectoral fin and the median fin fold, tend to persist during this stage, and appear even to have been adapted specifically for larval life [25]. Another general aspect associated with the transition into a larva is the slowing down of the overall rate of development, as well as the introduction of strong size and developmental differences between individuals. As a result, developmental progression can no longer be reliably predicted based on time alone. For instance, zebrafish develop synchronously up till feeding stages (5 dpf), but individual growth speed differs strongly afterwards. The appearance of developmental landmarks in larvae is therefore usually given as a function of standard length, rather than of time [22, 25, 31] (and see Fig. 14).

For the distinction between direct- versus indirect-development, we refer to “indirect-development” in species where the embryo transforms in a larva, and completes important aspects of the formation of its body plan during feeding stages. We refer to “direct-development” in cases where the embryo transforms directly into a juvenile, i.e. a “definitive phenotype” that possesses most aspects of the adult body plan, and lacks embryonic characters, such as the endoskeletal disk and the median fin folds. Therefore, indirect- versus direct-development is not related to the absolute speed of development, or to the relative sequence of appearance of developmental landmarks. Rather, the difference between these two developmental modes lies in the presence or absence of a larval period necessary for the completion of the body plan. We can illustrate this using the development of the pectoral fins in zebrafish (after the description by Grandel & Schulte-Merker 1998 [25]) and *A. burtoni* (this article) (Fig. 14). Both species progress through a very similar developmental sequence, namely formation of the endoskeletal disk (stage II), appearance of a median cleft in the disk (stage III), appearance of an additional dorsal and ventral cleft together with the first appearance anteriorly of distal radials and fin rays (stage IV), followed by a complete separation of the four fin radials as well as posterior generation of the full complement of distal radials and fin rays (stage VI) (stages given after Fig. 7). In *A. burtoni*, this sequence is completed within less than 1 week of development, between day three, with the first appearance of the endoskeletal disk, and day ten, when the fin has reached its definitive phenotype (Fig. 14). During this period (and in general before and shortly after onset of feeding), growth and development in *A. burtoni* are very homogenous and predictable (as also indicated by the low divergence in standard length, typical for direct-development (Fig. 14)).

In the indirect-developing zebrafish, the endoskeletal disk is also formed around day three post-fertilization, but has not yet started its further differentiation at the embryo to larva transition at 5 dpf, when feeding starts. After this point, the growth of the larvae slows down and becomes strongly heterogeneous (Fig. 14, standard length plot after Parichi et al. 2009 [22]). Hence, milestones in the development of the pectoral fins can only be reliably indicated with a reference to standard length [25]. From the embryo to larva transition on, the further differentiation of the endoskeletal disk is not only slower, with the fastest individual completing the formation of the pectoral fins only at about 3 weeks to 1 month [25], it also is heterogeneous to such an extent that individuals from the same clutch can be weeks apart in reaching the same developmental landmark. Such relatively slow and heterogeneous development can of course immensely complicate experimental approaches. In this sense, *A. burtoni* can function as a complementary direct-developing model system to circumvent the disadvantages associated with indirect-development.

These observations on direct- and indirect-development of course leave the issue of metamorphosis in *A. burtoni* unaddressed. Considering the universal presence of a metamorphic step in vertebrates and teleosts [4, 93, 94], it is likely that some form of cryptic metamorphosis is present as has been suggested for direct developing species [94], something which would be worth studying by investigating the underlying thyroid hormone pathways in *A. burtoni*.

### *A. burtoni* as a model system for evolutionary-developmental biology

**Fig. 15:**
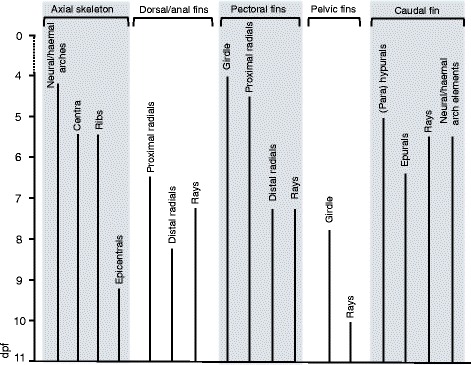
Overview of the sequence of development of the post-cranial skeleton of *A. burtoni*. Schematic representation of the development of the various morphological aspects described for the axial skeleton as well as the pectoral, pelvic, dorsal/anal and caudal fins, with the anatomical structures aligned along the X-axis and their development shown according to time (dpf) on the Y-axis. No timeline for craniofacial elements is included. Abbreviations: dpf: days post fertilization

Previous descriptions and investigations into the ontogeny of cichlid osteology have primarily focused on the development of the cranial skeleton [91, 95–97], and we are not aware of a detailed account of the ontogeny of the post-cranial skeleton in a *Haplochromine* cichlid species as given here. Our description of *A. burtoni* development shows that all of the adult skeletal features develop within 2 weeks after fertilization (most of them much earlier) (see Fig. 15), and the data reported here will give a useful reference for researchers striving to understand the development and evolution of the fish body plan using this system as a direct-developing alternative to zebrafish or medaka.

A wide range of fish species (predominantly teleosts) is now being developed as model resources [7, 98]. Many of these fishes are selected for their phylogenetic position, rather than for their suitability to the wet-lab. Cichlid fishes have been in the spotlight for several decades because of their spectacular radiation, and their rapid evolution and speciation. In addition, *A. burtoni* has already acquired a place as a model system in neuro-behavioral and physiological research [99–119]. Although this species has many traits that would make it a useful developmental system, it does not seem to have caught the eye of embryologists yet. Husbandry of *A. burtoni* is straightforward, it has a relatively high quality genome sequence available [35], and the possibility for microinjection into the fertilized egg makes it tractable to genetic strategies, such as transgenesis [120] and gene editing [39]. In our laboratory, we obtain high efficiency F0 mutation using CRISPR/Cas9, and we reach generation times of between 3 and 5 month.

## Conclusions

We show here that the complete osteology of *A. burtoni* develops within a time span of less than 2 weeks, an aspect in which it differs strongly from established model fish model systems, such as zebrafish and medaka. This rapid development from egg to free swimming juvenile fish offers scope for different experimental approaches than in these other teleost. Together with its experimental tractability, this will make it a suitable system for studies on the fish body plan. Its further development may prove a great help in addressing the evo-devo of the truly spectacular radiation of teleost fishes.

## Methods

### Material investigated

For adult skeletal preparations nine fish were fixed in 4% buffered PFA, bleached using H_2_O_2_ in 1% KOH solution and transferred to 100% EtOH. For skeletal stains, samples were incubated for several days on a shaking platform at room temperature in 0.004% Alcian blue in 70% EtOH/30% glacial acetic acid. Subsequently they were washed overnight in 70% EtOH/30% glacial acetic acid to reduce background staining. Afterwards, fish were stained in 0.04% Alizarin red in 1%KOH followed by further clearing using enzymatic digestion with trypsin in saturated borax solution. Embryos and juvenile fish were treated similarly except that these were not digested using trypsin. For each stage reported at least 6 embryos for the same time point (+/− 6 h) were examined.

### In situ hybridization

In situ hybridization was performed according to Woltering et al. 2009, 2014 [10, 121] with the difference that proteinase K treatment used was 8 min for 5 dpf, 10 min for 7 dpf and 12 min for 9 dpf. The *collagenX* probe (genbank locus: XM_005931031.2) was amplified from cDNA and cloned into the pGEMT vector (Promega) using primers FW: GTGAAAAAGGAAATGGAGGTGC, RV: TCACGTTGAAGCGATGAGGAAC.

### MF20 immuno staining

Immuno-histological stainings for muscle sarcomeres were carried out using the MF20 antibody [65] obtained from the Developmental Studies Hybridoma Bank, Iowa. Briefly, embryos were fixed in MeOH at − 20 °C overnight and stained with Alcian blue as described above. Embryos were rehydrated to PBS, incubated in PBS 0.01% Triton X1000 for 2 h, washed 5 times for 5 min in PBS, blocked for minimally 2 h in PBS 10% heat inactivated lamb serum and incubated overnight at 4 °C with a dilution of 1/200 MF20 primary antibody in PBS supplemented with 10% heat inactivated lamb serum and 0.01% Tween 20. The next day embryos were washed a minimum of 5 times for 1 h at room temperature with PBS supplemented with 0.01% Tween-20 and subsequently incubated with secondary antibody (Goat-anti mouse., DyLight 488, Thermo Fisher Scientific #35502, diluted 1:200) overnight at 4 °C. The next day embryos were briefly washed in PBS 0.01% Tween-20 and imaged using standard fluorescence microscopy.

### Animal husbandry and embryo culture

*A. burtoni* were kept in mating groups of 10–25 individuals at ~ 25 °C in the animal facility of the University of Konstanz. After collection, embryos were cultured in 100 ml plastic petri dishes in a 28 °C incubator in tapwater supplemented with 0,01μg/ml Methylene blue and penicillin/streptomycin (Sigma P4333) diluted 1:1000. To mimic the constant movement embryos would experience in a females buccal cavity, embryos were kept on a slow moving orbital shaker until 6 dph as the circulation at these stages seems to increase survival. After day 7 when embryos start to swim actively they were removed from the shaker. Particularly during pre-hatching stages it is essential to clean dishes from dead and morbid embryos as these can easily lead to infections of the whole clutch. Standard lengths (from the base of the caudal fin rays to the operculum) were measured on 10 embryos/juveniles a day from different clutches.

## Abbreviations

dpf: Days post fertilization
hpf: Hours post fertilization
